# Bringing cell therapy to tumors: considerations for optimal CAR binder design

**DOI:** 10.1093/abt/tbad019

**Published:** 2023-09-12

**Authors:** Richard Smith

**Affiliations:** Department of Research, Kite, a Gilead Company, 5858 Horton Street, Suite 240, Emeryville, CA 94070, USA

**Keywords:** cell therapy, CAR architecture, binder

## Abstract

Chimeric antigen receptor (CAR)-T cells have revolutionized the immunotherapy of B-cell malignancies and are poised to expand the range of their impact across a broad range of oncology and non-oncology indications. Critical to the success of a given CAR is the choice of binding domain, as this is the key driver for specificity and plays an important role (along with the rest of the CAR structure) in determining efficacy, potency and durability of the cell therapy. While antibodies have proven to be effective sources of CAR binding domains, it has become apparent that the desired attributes for a CAR binding domain do differ from those of a recombinant antibody. This review will address key factors that need to be considered in choosing the optimal binding domain for a given CAR and how binder properties influence and are influenced by the rest of the CAR.

## Teaser

Statement of Significance: Chimeric antigen receptor (CAR) cell therapy is transforming the practice of immunotherapy. This review will describe the key factors that must be considered in the generation and characterization of antibody-derived binders and their subsequent engineering into optimal CAR architectures.

## INTRODUCTION

**Figure 1:**
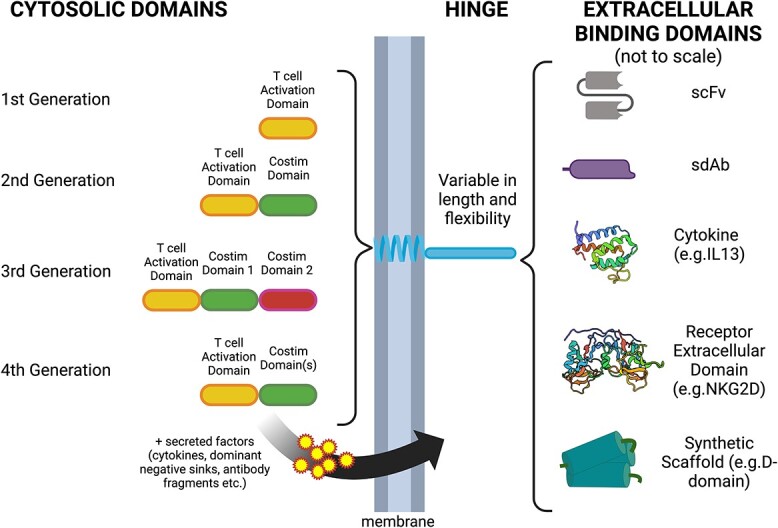
The modular architecture of chimeric antigen receptors. The various generations of CARs are defined by their internal domains. First-generation CARs consist of an antigen-binding domain fused via a hinge to the transmembrane and activation domain from CD3ζ. Second-generation CARs use a variety of hinges and transmembrane domains, plus a single costimulatory domain in addition to the antigen-binding domain and CD3ζ activation domain. This enables them to recapitulate the two signals required for full T-cell activation. Third-generation CARs build on the basic structure of the second-generation CAR by adding a second costimulation domain. Fourth-generation CARs are defined as having additional enhancing factors, such as cytokines, dominant negative receptors and antibody fragments, for example, that can promote increased potency and/or persistence. The hinge and transmembrane domains determine how the CAR interacts with other proteins in the cell membrane, with the optimal length and flexibility of the hinge being dependent on the epitope for the CAR’s binding domain. Representations of the classes of binders are shown not to scale, including scFv, sdAb, cytokines (IL13 is shown as an example [81]), receptor extracellular domains (NKG2D is shown as an example [75–78]) and synthetic scaffolds (the three-helix bundle D-domain is shown as an example [89, 90]). Created with BioRender.com. Cartoon representations of IL13 [178] and NKG2D [179] were derived via BioRender.com from the PDB database.

Chimeric antigen receptor (CAR)-T cells have shown impressive success in the clinic, with six approved therapies across a range of hematologic malignancies [1] (Table 1). CAR technology is being extended across other cell types, notably natural killer (NK) cells [2, 3] and macrophages [4], as well as a broader range of both liquid and solid malignancies and non-oncology indications [5]. All CARs consist of an extracellular domain containing a binding domain that recognizes a target cell antigen and a hinge that presents the binding domain on the surface of the engineered cell. A variety of hinges have been employed, derived from native proteins such as CD28, CD8α and IgG4. In addition, synthetic linkers can be used to add varying degrees of flexibility or rigidity. The extracellular domain is linked via a transmembrane domain to the intracellular signaling domains. These have evolved through the development of CAR technology (Fig. 1). In principle, CARs recapitulate the signaling that is driven by the endogenous T-cell receptor (TCR) complex, after the TCR recognizes its cognate major histocompatibility complex (MHC)–peptide complex on a target cell [6]. The TCR consists of two subunits, α and β, that recognize the MHC–peptide complex but do not themselves have signaling domains. The signaling domains are found in the associated CD3 chains: CD3γε and CD3δε heterodimers and CD3ζζ homodimers. CD3γ, δ and ε consist of a single extracellular immunoglobulin domain and a single cytosolic immunoreceptor tyrosine-based activation motif (ITAM) domain, while CD3ζ has a short unstructured extracellular domain and three cytosolic ITAM domains. First-generation CARs consisted of a binder:hinge extracellular domain linked to the CD3ζ transmembrane and cytosolic ITAM domains [7, 8]. While these CARs showed activity *in vitro* and in preclinical *in vivo* models, they failed to show efficacy in the clinic [9–11]. Subsequent generations of CARs looked to the broader mechanisms of T-cell activation to guide their design. While the primary signal driving T-cell activation is initiated by the TCR:CD3 complex, a second signal is also required for optimal activation [6]. Following engagement of the TCR with the MHC–peptide complex, a ring-like structure known as the immune synapse forms between the T cell and the target cell [12–14]. This structure contains additional signaling molecules, notably costimulatory molecules such as CD28 [15] and 4-1BB [16], which are responsible for initiating the second signal upon engagement with their cognate binders CD80 and CD86 (for CD28) and 4-1BBL (for 4-1BB) that are located on the target cell. Second-generation CARs include the cytosolic signaling domain from costimulatory molecules [17–21]. All approved CARs to date use either CD28- or 4-1BB-derived signaling domains. Other costimulatory domains, including those derived from OX40, CD2 and CD27, have also been investigated [20]. In second-generation CARs, the costimulatory domain is immediately proximal to the inner surface of the plasma membrane. Third-generation CARs attempted to stack CD28 and 4-1BB domains in a linear fashion in the hope of gaining advantages in activating both costimulatory pathways at the same time [22]. This has been a mixed success, presumably due to the ancillary signaling molecules needed to potentiate the signal not being able to reach the domain that is situated too far from the membrane [23]. An alternate approach sees parallel expression of two CARs in the same cell, targeting either the same antigen or two different antigens, with one CAR having the CD28 costimulatory domain and the other 4-1BB, enabling activation of both pathways upon target antigen engagement [24–28]. Fourth-generation CARs go beyond the CAR construct itself to include co-expression of an additional protein that can enhance activity and/or persistence of the cell or modulate the tumor microenvironment in order to overcome immune suppression within the tumor [29].

**Table 1:** Summary of CAR-T therapies approved by the US Food and Drug Administration

Name	Indication(s)	Target	Binder	Hinge/TM	Costimulation domain	Activation domain
Idecabtagene vicleucel	Multiple myeloma	BCMA	Murine scFv	CD8α	4-1BB	CD3ζ
Ciltacabtagene autoleucel	Multiple myeloma	BCMA	Dual VHH	CD8α	4-1BB	CD3ζ
Lisocabtagene maraleucel	Diffuse large B-cell lymphoma (DLBCL)	CD19	Murine scFv (FMC63)	IgG4	4-1BB	CD3ζ
	High-grade B-cell lymphoma					
	Primary mediastinal large B-cell lymphoma					
	Follicular lymphoma					
Tisagenlecleucel	DLBCL	CD19	Murine scFv (FMC63)	CD8α	4-1BB	CD3ζ
	Relapsed/Refractory acute lymphoblastic leukemia					
Brexucabtagene autoleucel	Relapsed/Refractory mantle cell lymphoma	CD19	Murine scFv (FMC63)	CD28	CD28	CD3ζ
	Relapsed/Refractory B-cell precursor acute lymphoblastic leukemia					
Axicabtagene ciloleucel	DLBCL	CD19	Murine scFv (FMC63)	CD28	CD28	CD3ζ
	Primary mediastinal B-cell lymphoma					
	High-grade B-cell lymphoma					
	DLBCL that results from follicular lymphoma					
	Follicular lymphoma					

CARs have also been introduced into NK cells and macrophages. As the binder in all cases is desired to drive tumor cell-specific engagement, the considerations for binder selection and design that will be discussed below are broadly similar across all cell types, though there are some differences in the rest of the CAR architecture that are becoming apparent. These will also be discussed subsequently.

## CONSIDERATIONS FOR BINDER DESIGN

In broad strokes, the considerations for identifying a suitable target for cell therapy in oncology are like those for a large molecule. Balancing differential expression between tumor and healthy tissue and considering the impact of on target/off tumor activity guide the establishment of a therapeutic window [30]. While the pharmacokinetics of cell therapy is different for that of a large molecule (as the cell therapy can expand and differentiate within the patient), the principles are largely similar [31]. In the case of B-cell malignancies, the approved CAR-Ts axicabtagene ciloleucel (Yescarta), brexucabtagene autoleucel (Tecartus), tisagenlecleucel (Kymriah) and lisocabtagene maraleucel (Breyanzi) all target CD19 via a binder domain derived from the same antibody, FMC63, resulting in collateral B-cell aplasia [32]. However, this is something that physicians can manage effectively. In contrast CARs targeting Her2 [33, 34], CEA [35] and CAIX [36], while showing promise preclinically in some instances, led to adverse outcomes in patients as a result of on target/off tumor activity. What factors need to be considered in identifying an optimal binder?

## CHOICE OF SCAFFOLD

Most CARs to date have relied on binding domains derived from the variable regions of monoclonal antibodies, generally reformatted as single-chain variable region fragments (scFvs). The scFv format permits facile reformatting of antibodies derived from conventional immunization campaigns. These are almost entirely, to date, raised against the extracellular domains of cell-surface proteins that are identified as viable tumor targets, though there are recent developments in generating scFvs that recognize intracellular tumor antigens with limited MHC restriction (which is a limiting factor in TCR-T cell therapy) through display technologies [37, 38] or using structural analysis to drive rationale engineering to optimize the binder [39, 40]. It remains to be seen how broadly applicable and indeed successful this approach will be. Additionally, scFv libraries have been used with various panning technologies to successfully generate CAR binders [41–44]. scFvs in CARs typically have a conventional structure with the heavy- and light-chain variable regions (VH and VL, respectively) joined by a flexible linker. The length and sequence of this linker are important in allowing stable association of the VH and VL domains [23]. Typically, G_4_S multimers are used, most frequently as a (G_4_S)_3_ 15-mer. The Whitlow 218 linker has also been used successfully, with one study showing that use of this linker increased binder affinity [45], though other studies have shown no difference in scFv binding properties when different linkers are used [46]. The optimal orientation of VH and VL domains is binder dependent [47–51]. Not all antibodies can be successfully reformatted to scFv. For isolated scFvs, unsuccessful conversion can manifest as low to no expression, or, if expressed, the scFv could aggregate [52]. Successful conversion as an independent scFv does not necessarily guarantee that it will form a functional CAR [46]. Not only could a given scFv negatively impact CAR expression, but it could also lead to target-independent, or ‘tonic’, signaling with concomitant exhaustion of the T-cell product because of scFv-driven clustering of the CAR in the T-cell plasma membrane [53, 54]. While this may seem to be a property to avoid, there is evidence that a tendency toward antigen-independent activation may actually yield a more potent CAR, with engineering strategies used to favor this such as manipulating the length of the linker between VH and VL domains [55]. This correlates with observations of tonic signaling in native, unmodified T cells being necessary to maintain antigen responsiveness, though it is important to note that in this case, the tonic signaling is driven from the TCR and is likely to be qualitatively different in terms of downstream mediators engaged when compared to CAR-driven tonic signaling [53, 56]. In addition to suboptimal binder design, tonic signaling can also be driven by overexpression of the CAR, which can be negated by using a weaker promoter [57]. Additional architecture changes through using different costimulation domains to limit exhaustion post-tonic signaling [58] or through optimizing the hinge to limit antigen-independent aggregation [59] can also be used to limit unwanted tonic signaling. In these instances, the optimal configuration of CAR and vector were determined empirically, highlighting the need for careful assay design to support CAR engineering.

While many CARs have utilized scFvs as their binding domain, several alternate scaffolds have also been used that potentially confer advantages over the scFv format (Fig. 1). Single-domain antibodies (sdAbs) derived from ‘heavy chain only’ antibodies that naturally occur in camelids (termed VHH domains) [60, 61] and sharks (termed VNAR domains) [62] are attractive as they offer the specificity of scFvs with a smaller size (12.5 versus 25 kDa). The reduced size is advantageous when considering the limited carrying capacity of the retroviral (8 kb) and lentiviral (10 kb) vectors that are typically used to deliver CAR-expressing constructs to T cells, as this frees up space in the vector to accommodate additional components to enhance the CAR-T cell’s potency and/or persistence [63]. While the VHH scaffold is unique to camelids among mammalian species, it is possible to humanize these domains [64] in an effort to reduce immunogenicity, though the potential to increase aggregation may confound this. Typically, this process may include CDR grafting onto existing humanized frameworks or homology modeling to identify similar human frameworks and residues that could be changed to increase the human sequence content without compromising the stability and specificity of the VHH domain. Engineering individual binders to be more human-like is not guaranteed to result in a stable, active binder. Therefore, display approaches, enabling selection of binders with both stability and the desired binding properties, are favored. Both synthetic humanized camelid VHH libraries [65, 66] and ‘camelized’ human VH libraries (where residues that form the hydrophobic interface between VH and VL domains in human antibodies are changed to their hydrophilic equivalents from camelids [67]) have been used successfully. It should be noted that binders generated in this way typically have lower affinity than conventional antibodies, presumably due to the phage selection process having a significant avidity component coupled with the relatively small surface area of the VHH binding interface. The subsequent development of transgenic platforms in both mice and rats, where human VH gene segments are knocked in and the endogenous rodent VH gene segments and CH1 exon are knocked out, have enabled the relatively simple generation of high-affinity single-domain binders through immunization that have been used as both recombinant proteins and in the context of CAR-Ts [68, 69].

The marketed CAR-T ciltacabtagene autoleucel (Carvykti) utilizes two nanobody domains that recognize distinct non-overlapping epitopes on BCMA, forming a biparatopic binding domain, and has shown positive results in the clinic [70–73]. The dual binding domains enhance avidity relative to a single binding domain, enabling a single CAR to bind to two target proteins, enhancing the formation of a robust immune synapse. Notably, the doses required for complete remission are 10-fold lower with the dual-nanobody CAR versus that required with a single-nanobody CAR. It remains to be seen if this observation can be generalized across other targets, but nevertheless, this speaks to the potential of non-conventional binders.

Binding domains from native proteins have also been successfully used in CARs, where the tumor-specific or tumor-enriched target is the cognate receptor of the ligand used in the CAR. A comparison of anti-CD70 CARs using a conventional second-generation architecture including an anti-CD70 scFv with an engineered variant of the native ligand of CD70, CD27 (engineered to include the CD3ζ activation domain at its cytosolic C-terminus) was performed by Sauer *et al*. [74]. CAR-Ts expressing the CD27:3ζ variant had superior antitumor activity *in vitro* and *in vivo* relative to the anti-CD70 second-generation CARs. The authors postulate the possibility that it is possible that many of the anti-CD70 CARs had sub-optimal architectures leading to tonic signaling, while the CD27:3ζ chimera has the optimal structure for engagement with its ligand in trans. Conceptually this suggests a more rapid route to generating a CAR-T where the target has a native membrane-associated ligand, thus negating the requirement for a binder generation campaign.

As the NK-cell receptor NKG2D recognizes a range of ligands that are expressed on most types of tumors, the NKG2D extracellular domain has been incorporated into CARs for use in both T- and NK cells [75–78]. This gives the CAR-transduced cells a more potent anti-tumor effect than that seen with native NK cells and has resulted in several assets reaching the clinic. However, NKG2D expression is not entirely restricted to tumors and on target/off tumor toxicity coupled with low efficacy has limited the clinical success of this strategy [79, 80], highlighting the need for tight specificity and control of CAR expression.

An additional advantage in using a native ligand is that high-resolution structural information describing the ligand:receptor interaction may be available, enabling rational engineering to manipulate affinity and specificity, thus avoiding the need to screen multiple novel binders or resort to affinity maturation. CARs using a cytokine as the binding domain have been termed ‘zetakines’. An IL13 zetakine has been utilized to target glioblastoma via IL13Rα2 [81]. The specificity for IL13Rα2 (which is glioma-restricted) over the more broadly expressed IL13Rα1 is driven by a point mutation (E13Y) that gives a 50-fold higher affinity for IL13Rα2 and a 5-fold lower affinity for IL13Rα1/IL4Rα over wild-type IL13. Similarly, an IL3 zetakine was designed to target CD123 in AML [82], with a second-generation construct being mutated to lower the affinity for CD123, thus enabling a broader distinction between low CD123–expressing healthy tissue and high CD123–expressing tumor cells. While utilizing endogenous ligands and binding partners can accelerate CAR generation, there is potential for the native binding protein to compete with the CAR for the target. Thus, generating a novel binder enables access to the entire surface of the target protein, which may include tumor-specific or tumor-enriched epitopes resulting from splice variants [83] or aberrant glycosylation [84, 85]. Non-antibody-derived binding proteins may confer additional advantages in terms of having smaller size (thus taking up less space in the vector), increased stability or by having binding surfaces that could interact with different epitopes on the desired target. A wide range of such proteins with antibody-like affinities, including adnectins, affibodies, avimers, anticalins and ankyrin repeats, have been developed for a range of applications [86] including CARs [87, 88]. The search for optimal scaffolds has extended to *de novo*-designed three-helix bundles, termed D-domains [89, 90], which have shown promising results when incorporated in CARs both preclinically [91] and clinically [92].

Early generation CARs, as with monoclonal antibodies, utilized scFvs derived from rodent antibodies. Indeed, the four approved anti-CD19 cell therapies all utilize an scFv derived from the mouse antibody FMC63 [93]. There are concerns that a non-human sequence could drive an anti-drug antibody response, leading to accelerated clearance of the therapeutic and preventing the potential for repeat dosing. This also potentially includes the junctions between domains in these chimeric molecules that could create novel epitopes, as well as other non-native scaffolds used as binding domains [94]. Data from the ZUMA-1 trial, testing axicabtagene ciloleucel (Yescarta), identified three patients who developed antibodies against murine IgG sequences, though these did not appear to significantly impact drug exposure [95]. Clinical studies with tisagenlecleucel (Kymriah) included a patient population with a very high incidence of existing anti-murine IgG antibodies (86% of patients), though this too did not appear to negatively impact efficacy [96]. It is hypothesized that the lymphodepletion regimens used prior to infusion of both anti-CD19 CAR-T therapies limits the potential for generating a neutralizing immune response. It should also be noted that the B-cell aplasia that generally occurs post-CD19 CAR-T infusion mitigates the generation of new antibodies. Anti-CD19 CARs utilizing human binders are currently under clinical development and have demonstrated efficacy in patients who previously relapsed or failed to respond to a murine anti-CD19 CAR-T [97–100]. It is also important to note that even when using fully human components, the chimeric nature of CARs does yield novel, potentially immunogenic sequences at the junctions between the domains in the CAR [101]. In contrast, it has been shown in one trial with the anti-BCMA CAR-T ciltacabtagene autoleucel that anti-CAR antibodies were detectable in six out of seven patients who relapsed, while only one out of eight patients with ongoing response had detectable anti-CAR antibodies. The development of anti-CAR antibodies is considered a significant risk factor for relapse [102]. It should be remembered that the CAR in this case uses dual llama-derived VHH binding domains. Resultingly, while the anti-BCMA CAR can target plasma cells, it does not induce B-cell aplasia as seen with anti-CD19 CARs, which may explain the induction and maintenance of a humoral response.

Anti-CAR antibodies have been detected against other murine-derived binding domains including the generation of anti-idiotype antibodies that could antagonize target engagement. Humanization of murine antibodies does not necessarily solve this problem, as residual murine framework sequences retained to maintain function can still drive immunogenicity as in the case of an anti-TAG72 CAR [101]. There has been one case reported of an IgE response to an anti-mesothelin CAR that led to fatal acute anaphylaxis [103]. Cellular responses have also been detected against a carbonic anhydrase IX CAR, with patient PBMCs collected after at least two rounds of CAR-T infusion responding to irradiated CAR-T cells [104]. Cells from several patients were shown to respond to peptides derived from the VH and Vκ domains used in the CAR. It should also be noted that PBMCs from other patients were shown to respond to epitopes derived from the retroviral vector used to transduce the CAR-T cells. While many of the design considerations used to reduce potential immunogenicity for protein therapeutics are applicable for CARs, the influence of additional non-human proteins such as the viral vector or gene/base editing systems (if these are being used) do add additional complexity and necessitate careful consideration when moving into the clinic [105].

## AFFINITY AND AVIDITY

As previously noted, CARs endeavor to recapitulate T-cell activation as induced by the TCR/CD3 complex. TCRs typically have low affinities for the cognate MHC–peptide complex, with Kds typically in the order of 10^−4^–10^−6^ M. TCRs can recognize less than five targets per cell, though it is apparent that a single MHC–peptide complex can serially engage with hundreds of TCRs, building the immune synapse and amplifying signal one. It has been shown that high-affinity TCRs do not improve efficacy, supporting the notion that sequential brief contacts are required for optimal T-cell activation [106]. In contrast, CARs use binders that typically have higher affinities than TCRs, in the order of 10^−6^–10^−9^ M [107]. It is important to consider that the mechanism of activation via CAR differs from a native TCR, in that signal one (via the CD3ζ ITAMs) and signal two (via the costimulatory domain) arise from the same polypeptide and are triggered by the same antigen recognition event. In contrast, when a native TCR is engaged, signal two arises from a costimulatory receptor:ligand interaction separate from the TCR. This also reflects differences in the structure of the immune synapse between TCR and CAR activation. A natural immune synapse is characterized by concentric rings of defined clusters of proteins centered on the TCR in the central supramolecular activation cluster (SMAC) that also includes protein kinase C-θ, CD4, CD8, the costimulatory receptors CD2 and CD28 and the cytosolic signaling mediators Fyn and Lck. This is surrounded by the peripheral SMAC, containing adhesion molecules such as LFA1 and cytoskeletal proteins that stabilize the structure and further out the distal SMAC, containing regulators such as CD45 [14]. In contrast, CAR-mediated synapses are disorganized, with punctate distribution of the CAR and associated costimulatory and adhesion molecules [108–110]. This aberrant organization can reduce the quality and duration of the activating signal driven by the CAR. Majzner *et al*. [111] demonstrated that the quality of the immune synapse is more influenced by the hinge domain than the binder, though there are considerable co-dependencies between the hinge and binder domains. Optimizing the structure of the CAR extracellular domain and considering how the target protein on the tumor cell fits into the synapse are increasingly being seen as important considerations when designing CARs [108, 110].

The optimal affinity for a CAR binding domain is dependent on the extent of differential target expression between the tumor and normal tissue. The optimal affinity should drive location-appropriate activation. It has been demonstrated that CAR-Ts have different activation thresholds for cytokine production, proliferation and cytotoxicity, which must be factored into determining binder affinity. Increasing affinity for the target antigen will eventually cause maximal T-cell activation while decreasing discrimination for off-tumor cells. Tuning binder affinity has been shown to increase the differential between tumor and normal tissue for a number of targets such as EGFR [112] and ErbB3 [113]. Lower affinities have the potential to separate out therapeutic potency from toxicity. A novel CD19 CAR, CAT19, has a binder with a Kd for CD19 of 14.38 nM, compared to FMC63, which has a Kd of 0.328 nM [114]. This difference in affinity was driven primarily by CAT19 having a relatively faster off rate compared to FMC63. In a Nalm6 model CAT19, CAR-T cells showed increased potency relative to FMC63 CAR-T cells, where both CARs utilized CD8α hinge and transmembrane domains, 4-1BB costimulation domains and a CD3ζ activation domain. In particular, the CAT19 CAR-T cells showed greater sensitivity to low-antigen target cells. The CAT19 CAR-T has been studied in the clinic (CARPALL, NCT02443831) and showed similar activity to tisagenlecleucel (the commercial anti-CD19 CAR-T product with similar architecture), though with much reduced toxicity [115].

An alternative route to modulating affinity is to perform alanine scanning across an existing binder, as performed by Halim *et al*. [116] with FMC63, deriving a series of lower-affinity binders that retained specificity for CD19 while maintaining avidity. In terms of avidity, it is also important to remember that additional contacts beyond the CAR and target occur between the T cell and the target cell, which also can support CAR-T activation. Many of these interactions occur during engagement between a T cell and a normal antigen-presenting cell, promoting both costimulation and checkpoint inhibition. Some of these interactions have been shown to be clinically important. For example, the T-cell costimulatory molecule CD2 plays a role in migration, function and survival of T cells, with CD2 activation making T cells less vulnerable to PD1-mediated exhaustion [117, 118]. It has been shown that tumors with higher expression of the CD2 ligand, CD58, are more readily cleared. These observations highlight the importance of understanding the profile of costimulatory molecules on the surface of target cells, particularly when choosing physiologically appropriate assay cell lines during development of novel CARs.

While reducing binding affinity can expand differential binding between high and low target-expressing cells, it comes with the risk of reducing the gap between target-specific and non-specific binding. An alternate approach is to focus on modulating avidity. Ma *et al*. devised a combinatorial library approach utilizing a CAR-dependent reporter system that enabled selection of CARs with greater differential binding for CD38-high expressing cells over CD38-low expressing cells [119]. For comparison, a high-affinity anti-CD38 CAR that was used to develop the system was unable to differentiate between high- and low-expressing target cells. Successive rounds of deselection of binders to CD38-low cells alternated with selection on CD38-high cells yielded binders with the desired properties. The lead novel binder had 10-fold lower affinity than the original high-affinity binder (Kds of 8.6 versus 0.65 nM) yet had the same potency and efficacy as the high-affinity binder versus CD38-high target cells while showing limited efficacy against target cells expressing normal physiologic levels of CD38. Avidity can also be increased by increasing the valency of the CAR. This is postulated to be the mechanism by which ciltacabtagene autoleucel has increased efficacy versus competitor anti-BCMA CARs, with the CAR containing two VHH domains that bind distinct epitopes on BCMA. This confers greater sensitivity to enable targeting of cells with low BCMA expression [70–73]. This mechanism is likely to be limited to targets where expression on healthy tissue is very low or absent or where on target/off tumor targeting (as in the case of CD19 in B-cell malignancies) can be clinically managed.

While technologies for measuring affinity of protein:protein interactions such as surface plasmon resonance and biolayer interferometry are widely used and understood, there is a still a lack of equivalent quantifiable measures of avidity between two cells as in the CAR-T:tumor cell interaction. Acoustic force spectroscopy is being increasingly used to determine the amount of force, in the form of ultrasound waves, that are required to dislodge CAR-T cells from a monolayer of target cells, with the amount of force needed being proportional to the strength of interaction. This platform has been used to distinguish between a BCMA-specific CAR and an APRIL-specific CAR that had similar affinities for their respective targets, but differing *in vivo* and clinical responses, with the APRIL-specific CAR performing worse. Acoustic force spectroscopy indicated weaker target cell binding by the APRIL CAR-Ts suggesting that these CARs were not able to form as effective synapses as the BCMA CAR [120]. This gives the potential for a relatively straightforward *in vitro* assay that could be predictive of *in vivo* and clinical CAR activity. However, predictability may be target dependent as there are publications that suggest CARs with the highest avidity as measured with this system have the best *in vivo* activity [24] while others describe an intermediate ‘optimal’ avidity [116]. Clearly, there are additional factors that need to be determined on a case-by-case basis for this assay to be considered truly predictive, but this technology nevertheless provides additional intriguing data to aid in CAR selection.

While optimizing affinity and avidity, it is critical to understand antigen expression across all appropriate tissues and tumors, particularly given that heterogenous antigen expression is a common characteristic of solid tumors. This can be addressed by targeting multiple antigens simultaneously, or by controlling expression of a more broadly, yet more consistently expressed antigen to within the tumor using logic gated systems. Additionally, the level of CAR expression on the CAR-T cell should be considered. Typically, high expression of the CAR is desired [121]. In a normal T-cell response, the TCR is downregulated after engagement with the cognate MHC–peptide complex, a mechanism by which the immune response is carefully moderated. This has also been observed with CARs designed to target T-cell malignancy antigens [122], where the CAR-Ts have been shown to ‘auto-tune’ low-affinity CAR expression by lowering CAR expression, thus reducing fratricide. This also broadened the therapeutic window on non-T cells, in which Epstein–Barr Virus transformed B cells with enhanced HLA-DR expression (which is also expressed on T cells) were killed preferentially over normal B cells.

## CO-DEPENDENCIES BETWEEN BINDER EPITOPE AND HINGE DOMAIN

**Figure 2:**
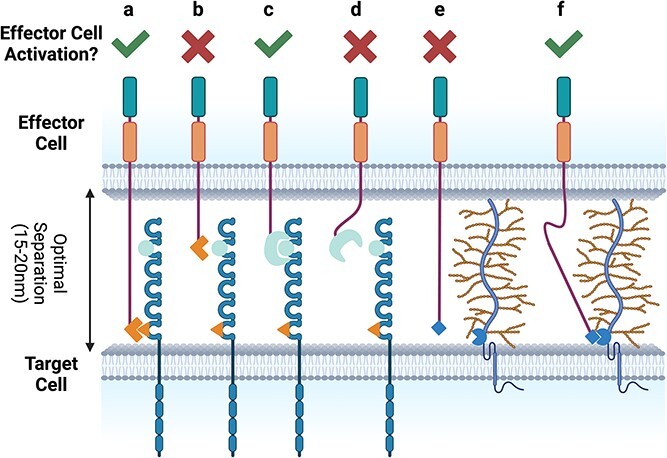
Relationship between hinge length and flexibility and binder epitope. Optimal T-cell activation requires maintenance of a gap of 15–20 nm between the T cell and the target cell. This enables recruitment of all the components of the immune synapse to the point of cell:cell contact. For CARs targeting membrane distal epitopes (A), a long hinge is required. The same binder on a short hinge will not enable optimal T-cell activation (B) as it will be unable to reach it epitope. Conversely, a membrane-distal epitope can be effectively engaged by a CAR with a short rigid hinge (C), while a more flexible linker may not thermodynamically favor binding [127, 128] (D). A rigid hinge was found to limit access to a membrane-proximal epitope in the bulky glycoprotein MUC1 (E), while inclusion of a flexible region in the hinge enabled effective binding with the target epitope [129] (F). Created with BioRender.com.

As we have seen, the formation of an effective immune synapse is critical for CAR-T activity. The synapse requires the T cell and target cell to be separated by approximately 15–20 nm [123, 124], allowing optimal engagement between the TCR and MHC–peptide complexes, as well as assorted costimulatory interactions. Intuitively, one would assume that a CAR extracellular domain should, for optimal activity, fit into this space. This requires a co-dependency between the epitope of the binding domain and the length and flexibility of the hinge (Fig. 2). Numerous studies have shown that effective targeting of membrane-proximal epitope requires a hinge that is long enough to enable the binding domain to reach its epitope while maintaining the optimal distance between the T cell and the target cell. McComb *et al*. [125] identified a series of camelid nanobodies targeting EGFR that resulted in CARs with high potency against both high- and low-target expressing cells. These CARs utilized a CD8α hinge; sequential truncation of this hinge attenuated signaling in the resulting CAR-T cell but increased selectivity for high-target expressing cells over low-target expressing cells, thus indicating a potential strategy for differentiating between tumor and normal tissue. Epitope location was also shown to be a critical determinant in choice of hinge domain in the same paper. CARs utilizing a binder recognizing a membrane-proximal epitope on Her2 were shown to have reduced activity when their hinge was truncated, while CARs using a binder that recognized a membrane-distal epitope in EGFRVIII were unaffected by hinge truncation. It is hypothesized that even with a truncated hinge, the latter EGFRVIII-targeting CARs were still able to support formation of a viable immune synapse given the accessibility of the epitope, while the truncated anti-Her2 CARs were unable to maintain the optimal distance between the target cell and the T cell to enable synapse formation. The importance of maintaining the optimal distance between the T cell and the target cell was highlighted by Xiao *et al*. who showed that using combinations of binder and hinge that created too much space between the cells on either side of the synapse was suboptimal, as it allowed the phosphatase CD45 to enter the synapse and shut down signaling [126].

While the length of the hinge is clearly important, the relative flexibility or rigidity of the hinge can influence CAR activity. Rigid IgG4-derived short hinges have been shown to be more effective than more flexible CD8α-derived hinges when targeting membrane-distal epitopes (Fig. 2C and D) [127, 128]. Conversely, a more flexible linker derived by inclusion of the IgD hinge between the scFv and CD28 hinge gave increased potency to an anti-MUC1 CAR over the parent CD28-only hinge, despite the more flexible CAR having lower expression [129]. MUC1 is a bulky glycoprotein, and increased flexibility presumably enables the binder to better access the membrane-proximal epitope (Fig. 2E and F).

The length and flexibility of the hinge are clearly critical for enabling formation of a productive immune synapse, but does this region of the CAR have any properties beyond merely providing structure? The most widely used hinges are derived from immunoglobulin (Ig) family proteins. Ig domains are stable, approximately globular structures that are frequently found in the extracellular domains of membrane proteins where they can mediate both structure and function. While Ig domains from IgG1 antibodies mediate engagement with Fcγ receptors as a critical property of this class of antibody, this makes them unfavorable for use as CAR hinges [130, 131]. In contrast, IgG4 antibodies do not bind to Fcγ receptors and have been successfully used as CAR hinges as in the case of lisocabtagene maraleucel [132]. Further efforts to derive a long hinge that does not drive unwanted biological effects have also explored Siglec-derived sequences [133] that showed better *in vivo* activity than comparable CARs using an IgG1-derived hinge. Alternatively, Ig domains from non-antibody proteins such as CD28 (axicabtagene ciloleucel and brexucabtagene autoleucel) or CD8α (tisagenlecleucel) have been widely used. While these were initially considered to be largely inert beyond their structural role, it has become apparent that the choice of hinge and transmembrane domain can profoundly impact CAR activity.

Fujiwara *et al*. [134] compared first-generation anti-VEGFR2 CARs utilizing CD28 and CD8α-derived hinge and transmembrane domains and showed that the CARs with the CD28 hinge had greater cytotoxic and cytokine-producing activity in mouse CAR-T cells than the equivalent CARs using the CD8α hinge. This observation was also seen in second-generation CARs against the same target, using a CD28 costimulation domain. Further work from the same group has indicated the differences in glycosylation, and the propensity for intermolecular disulfide bridging can influence antigen-dependent and -independent activity. Given that the parent molecules require clustering (induced by engagement with their cognate ligands), this should not be surprising. Dimerization of endogenous CD28 following engagement with its ligands, CD80 and CD86, drives the second signal required for T-cell activation. The costimulatory domain in a CAR generates an equivalent signal when the CAR engages with an antigen on a tumor cell. It has been demonstrated that CD28 hinge/transmembrane domain CARs can heterodimerize with native CD28 on the T-cell surface [135]. This increases the recruitment of native CD28 to the CAR, and thus the immune synapse, and may explain the stronger signal and increased sensitivity to lower antigen levels seen when using the CD28 hinge and transmembrane domains. Majzner *et al*. utilized confocal microscopy to show that anti-CD19 CARs (all utilizing the same binding domain) had similar distribution on the T-cell surface, irrespective of the hinge used, but that CD28 hinge/transmembrane domains induced faster synapse formation than CARs incorporating a CD8α hinge/transmembrane domain as determined by the recruitment of a ZAP70-red fluorescent protein fusion to the intracellular side of the synapse [111]. Additionally, CD28 hinge and transmembrane domains conferred greater sensitivity to lower antigen levels than both the IgG4 and CD8α hinges and transmembrane domains, suggesting higher potency in conditions where target expression may be lower. This has clinical impact in that when targeting a population of tumor cells with a range of target antigen expression even cells expressing low levels of antigen will be eliminated. For CD19, where on target/off tumor cytotoxicity results in the clinically manageable outcome of B-cell aplasia, this could provide a significant advantage in driving a complete response. However, many solid tumor targeting concepts rely on differential expression of antigen between healthy tissue and tumor important for establishing a therapeutic window. The binder itself could be engineered with a peptide mask that is only removed when exposed to the proteolytic milieu of the tumor; this is an approach that has been widely explored with recombinant antibodies [136]. An initial preclinical study with a masked anti-EGFR CAR demonstrated the feasibility of this concept in the context of CARs [137], though this has yet to be extended to other targets. It is likely that successful targeting of many tumors will require more advanced techniques such as targeting combinations of multiple antigens, using conditional logic gates to control when and where the CAR-T becomes activated. This is outside the scope of this review but has been discussed extensively elsewhere [138–143].

As mentioned previously, there is also extensive interest in using CARs in NK cells and macrophages, particularly in the targeting of solid tumors where these cell types may have a significant advantage over T cells. While considerations for identifying an optimal binder for a given target are generally cell-type agnostic, there are some cell-specific architectural features of note. While many CAR-NK constructs have used the same architectural components as CAR-Ts (reviewed extensively here [144]), use of transmembrane components taken from NK cell proteins such as DNAM1, 2B4 and NKG2D yields a more potent product [145]. Similarly with macrophages, many of the learnings from T cells have been shown to be transferable [146], though using macrophage-specific activation domains such as FcγR, MerTK and Megf10 has been shown to enhance cytotoxicity and phagocytosis [147]. These cases further reinforce that choice of binder needs to be considered holistically in the context of the rest of the CAR structure and the host cell, with physiologically relevant functional assays being employed to select the optimal architecture.

## UNIVERSAL CARS AND T-CELL ENGAGERS

**Figure 3:**
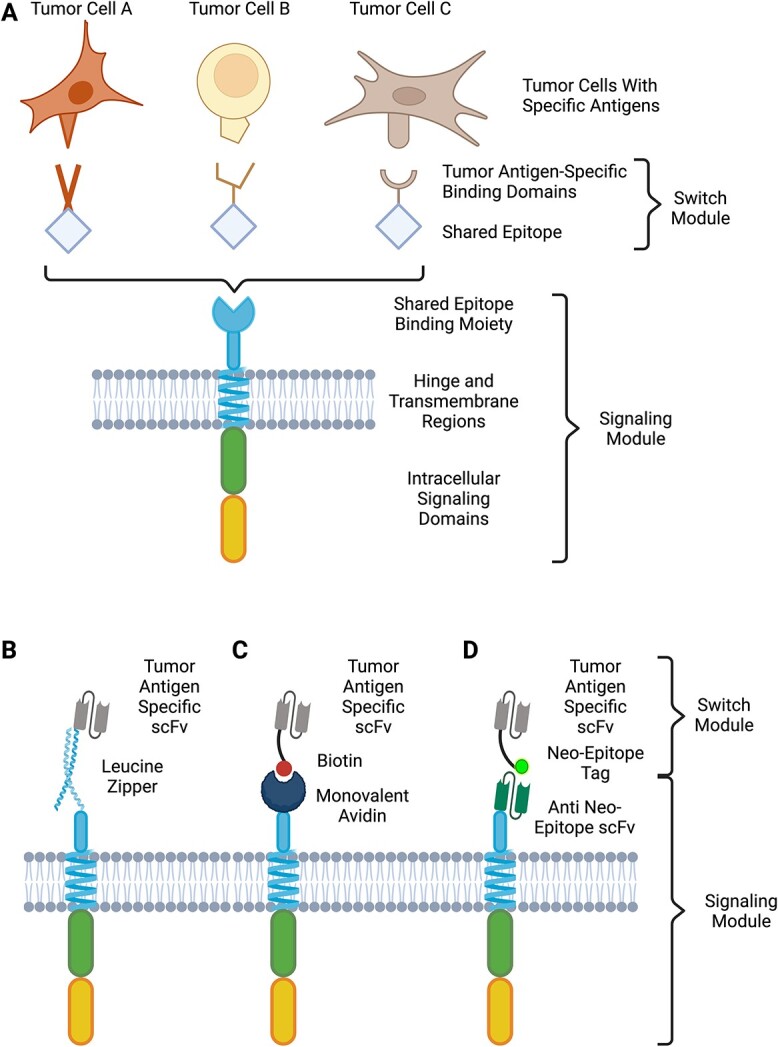
Universal CAR principles and designs. The Universal CAR concept consists of two segments: the signaling module that resembles a conventional CAR in its architecture and a soluble switch module (A). The switch modules are bispecific molecules with one domain that is shared across all switches and that is specific for the binding moiety on the signaling module. The tumor antigen-specific binding domain can be varied to enable targeting of different antigens and/or cell types. A range of different shared epitopes have been explored using this concept. SUPRA-CARs incorporate leucine zippers (B) with one half of the zipper forming the signaling module binding domain and its cognate partner being part of the switch module. Monovalent avidin (C) permits the use of biotin as the shared epitope on the signaling switch. Alternatively, the signaling module binding moiety can be an scFv that is specific for a neo-epitope that is not found in human tissues (D). Such neo-epitopes have included fluorescein or novel peptides. Created with BioRender.com.

While standard CAR-Ts as described have met with clinical success, their development and manufacture are complex. An increasingly common concept is that of the ‘universal CAR’, which couples a standardized cell product (either autologous or allogenic) with a bispecific switch module [148]. This switch module has one arm that binds to a signaling module that resembles a standard CAR on the manufactured T cell, while the other arm binds to a tumor-specific antigen (Fig. 3A). While the tumor-binding portion of the switch module is most readily generated by reformatting antibodies into Fabs or scFvs, alternate scaffolds, including VHH and peptides, have all been used [149–152]. A variety of approaches have been taken to enable the interface between the switch and signaling modules. The naturally occurring dimerization motif, the leucine zipper, has been used in the split, universal and programmable (SUPRA) CAR system (Fig. 3B) [152, 153]. In this case, optimization of the switch has not only included optimizing the tumor antigen binder but also tuning the affinity of the leucine zipper. This enables control over the relative interactions of simultaneously dosed switches (for example, if targeting two antigens with different expression levels), or enabling a high-affinity leucine zipper to be used to outcompete the tumor binders to shut down cell activation, thus serving as a safety switch. The interaction between biotin and avidin is highly specific, and the biotinylation of proteins is straightforward and scalable, making this interaction a viable approach for universal CARs (Fig. 3C) [154]. These biotin-binding immunoreceptor CARs have been shown to function both *in vitro* and *in vivo*, where presence of endogenous biotin has been shown to not compete with the switch module, nor activate the CAR. *In vivo* clearance of tumor from a human ovarian tumor mouse model was dependent on the presence of both the CAR-T cells and the switch module indicating the specificity of this system [155]. Alternate approaches have incorporated neo-epitopes that are not found in human tissues as the shared epitope in the switch module (Fig. 3D). This reduces the potential for cross-reactivity with endogenous proteins to either antagonize the interaction between signaling and switch modules or drive inappropriate activation of the signaling module. Fluorescein isothiocyanate (FITC) has been used as a ligand, with a cognate scFv serving as the binding partner on the signaling molecule. FITC has then been conjugated to scFvs targeting CD19 and CD22 to target B-cell malignancies. Advances in conjugation technology, including inclusion of non-natural amino acids to drive site-specific conjugation, enables tightly controlled, reproducible generation of the switch module. A wholly small molecule switch module has also been produced by linking FITC to folate, as the folate receptor is overexpressed on solid tumors [156]. Preclinical studies have shown that this small molecule switch can readily penetrate tumors and is cleared rapidly from receptor negative tissues. In addition, dosing with intravenous FITC to compete with the switch provides a potential ‘off-switch’ for CAR-T activation [157]. Novel peptides can also be readily incorporated as tags in a recombinant switch module, with a specific scFv being available for inclusion in the signaling module. Successful *in vitro* and *in vivo* studies have been performed with both the 10-amino acid 5B9 peptide [151], derived from an autoantigen known in Sjogren’s syndrome and systemic lupus erythematosus, and the 14-amino acid PNE peptide derived from the yeast transcription factor GCN4 [158]. It should be noted that while the 5B9 peptide is derived from an autoantigen, screening of sera from autoimmune patients has not revealed any competing antibodies [159].

In many ways, the mode of action of the switch module is conceptually like that of bispecific T-cell engagers that function the same way, bringing the T cell and the tumor cell together, but bind to the CD3ε chain of endogenous T cells rather than to an artificial receptor. This distinction is important when considering how the T cell subsequently signals. As discussed previously, the synapse between a CAR-T cell and the tumor cell is disordered rather than the neat bullseye structure seen in a TCR-mediated synapse [108–110]. Bispecific T-cell engagers have also been shown to induce the formation of more natural, bullseye synapses, presumably as the rest of the TCR is recruited to the complex as the bispecific T-cell engager binds to CD3ε [160]. The development of universal CARs needs to take the same considerations for synapse formation into account as conventional CARs, in this case with the added complexity of needing to accommodate switch modules that bind to a range of targets and epitopes while maintaining an optimal distance between the T cell and the tumor cell.

The universal CAR cell product can include additional enhancements to increase its potency and persistence over endogenous T cells and separates the cell product from the potentially immunogenic recombinant adaptor [161], though it does lock the development program into a single CAR architecture, necessitating careful design of the adaptor molecule to ensure it binds to its tumor antigen at an optimal epitope with appropriate affinity. Having a single-cell product manufacturing process greatly simplifies the targeting of multiple tumors, as the adaptor molecules can be generated using more mature large molecule development and manufacturing processes. There are additional complexities during clinical development as the adaptor molecule should have appropriate pharmacokinetic and biodistribution profiles that enable it to bring the universal CAR to the tumor in a clinically efficacious manner [148]; the current crop of clinical trials investigating the universal CAR concept will undoubtedly shed light on these questions [162]. More advanced concepts have leveraged developments in machine-learning enabled protein design to create switches that can perform logic on the cell surface. LaJoie *et al*. [163] developed the colocalization-dependent latching orthogonal cage-key protein, or Co-LOCKR, which uses a *de novo* designed cage protein to sequester a latch domain in an inactive conformation. Binding to a separate key protein releases the latch enabling engagement with an effector, such as a CAR-T cell. The Co-LOCKR system can be designed to target cells expressing two different antigens that distinguish the cells as being targets. Manipulating the affinities of the cage, latch and key domains can enable tuning to different relative levels of each antigen giving potential to have greater targeting control.

It may be possible for the CAR-T cell to be engineered to produce the adaptor protein *in situ*. Indeed, there have been several studies that have generated CAR-T cells that can produce bispecific T-cell engagers that target a second antigen [164–166]. The rationale for this concept is to deliver to the tumor a potent molecule that targets a highly expressed tumor antigen that may be expressed on healthy tissue, thus mitigating the potential for systemic toxicity. Additionally, this can also harness bystander non-engineered tumor infiltrating lymphocytes, broadening the scope of the therapeutic response. Using CAR-engineered cells as local protein factories has also been broadly used for delivering cytokines and other proteins that can modulate the tumor microenvironment to be more immunologically favorable to the cell therapy, a concept that is the foundation of fourth-generation CARs, and has been reviewed in depth elsewhere [167–170].

## COMBINATORIAL APPROACHES TO OPTIMIZING CAR BINDERS

Early in the history of cell therapy, existing antibodies were simply converted into scFvs and grafted onto a standard CAR architecture. While this can yield a functional construct (or indeed constructs as we look at the multiple approved architectures containing the anti-CD19 binder FMC63; Table 1), this may not be optimal for CAR-T activity, phenotype and persistence, particularly as we move into more complex systems where tumor penetration and using targets that are not completely tumor specific become challenges. As previously discussed, lower-affinity binders may enable better distinction between a tumor and healthy tissue, when the target antigen is more highly expressed on the tumor [112–115]. Unfortunately, conventional antibody screening techniques favor stronger binders; therefore, there has been a trend toward functional binder screening in the context of a CAR. A simple approach to reduce affinity is to perform saturation mutagenesis of the CDRs of an existing binder and screen for new binders that cover a range of reduced affinity and then test in a CAR format [116]. This has been used successfully to generate CD229 binders with reduced affinity that yield CARs that can better distinguish multiple myeloma cells from healthy lymphocytes that also express CD229, albeit at a much lower level [171]. A more rational approach was taken by He *et al*. [172], using cryo-EM structures of CD19 complexed with CAR binders FMC63 (used in all approved CD19 CAR therapies) and SJ25C1 (used in a number of clinical trials) to identify the residues that form the interface with the target and then performed molecular dynamics simulations to calculate the contribution of each residue. This informed the design and testing of a set of mutants with a range of affinities that yielded CARs with differentiated sensitivities to variable target antigen levels.

When developing a screening platform, it is important to have selection mechanisms and readouts that are both physiologically relevant and enable identification and recovery of positive hits. As described above, Ma *et al*. [119] optimized the affinity of CD38 CARs by successive rounds of functional screening and counter-screening against high- and low-target expressing cells purposely selected to match target expression in tumors and healthy tissue, respectively. Di Roberto *et al*. [173] developed a reporter cell line incorporating green fluorescent protein (GFP) under the control of the IL-2 promoter, thus serving as a marker of T-cell activation. A CAR library was inserted via CRISPR/Cas9 into the TCR locus (ensuring consistent expression across all library members). This system was successfully used to identify low-affinity variants of the binder derived from the anti-HER2 antibody trastuzumab that could better distinguish between high-expressing tumor cells and low-expressing healthy tissue than CARs derived from the parent antibody that had previously been shown to be toxic in the clinic [33, 34]. In this case, the GFP reporter enables fluorescence-activated cell sorting to allow isolation of cells carrying CARs that confer the desired properties. Ochi *et al*. [44] developed a screening system in primary human T cells where they generated libraries by splitting an existing tumor binder that was suboptimal as a CAR into two new libraries: the existing VH domain fused to a VL library and the existing VL domain fused to a VH library. Through extended stimulation of the CAR-T library by target-specific tumor cells, new CARs with enhanced specificity and activity were identified. This format enriches for CAR-Ts that can serially kill multiple target cells. It is noteworthy that one of the targets used was an MHC:peptide complex (A2/NY-ESO-1_157_), with the resulting CAR serving as a TCR mimetic that was highly specific to the presented peptide. In addition to using combinatorial approaches to screen for binders, there are considerable efforts to use similar systems to screen combinations of hinges and costimulatory domains [174–176], including novel sequences. Single-cell sequencing can enable deep phenotypic characterization, enabling comparison with transcriptional profiles of tumor-infiltrating lymphocytes [177]. This enables a library screen to go beyond a simple proximal readout (such as a reporter gene under control of an appropriate promoter) to identifying hits that induce complex biological responses.

## CONCLUSIONS

CAR-T cells have transformed immunotherapy, enabling a patients’ own cells to be reprogrammed to specifically target a tumor. In this sense, they go one step beyond bispecific T-cell engagers in that the engineered therapeutic only has to engage with the tumor cell, rather than cross-linking two cell populations that need to be adjacent, and with the therapeutic molecule present, for the therapeutic response to occur. While CARs frequently utilize antibody-derived binding domains, it is important to consider how the antibody binding domain will drive the function of this complex cellular product and what architecture will be used to present the binding domain at the cell surface. While much empirical work has been done to identify optimal architectures, in many of these cases, the field still needs to build equivalent data sets to those that have been acquired with monoclonal antibodies to enable effective understanding of structure–function relationships and rational design of CARs. Additionally, being able to identify what properties of parent antibodies favor conversion into a CAR format will simplify the selection of binders for inclusion in a CAR. High-throughput screening platforms, coupled with the development of physiologically relevant functional assays and tools to analyze complex data sets, will be essential in this effort. In particular, the field needs to develop a clear understanding of what assays and platforms will provide accurate *in vitro*/*in vivo* correlation to ensure that we can more rapidly identify viable candidates to move into the clinic. These will also be essential in supporting development of more complex products utilizing conditional regulation of CARs via sensory logic gates. At the same time, reverse translation will be vital in linking *in vitro* engineering to clinical outcomes, in terms of both efficacy and safety as cell therapy extends beyond its early successes in hematologic cancers to solid tumors and beyond oncology.

## ABBREVIATIONS

CAR; chimeric antigen receptor. TCR; T-cell receptor. MHC; major histocompatibility complex. scFv; single-chain antibody-variable region fragment. NK; natural killer. ITAM; immunoreceptor tyrosine-based activation motif. FITC; fluorescein isothiocyanate.

## Data Availability

This is not applicable.

## ANIMAL RESEARCH STATEMENT

This is not applicable.
