# Correction to: Bringing cell therapy to tumors: considerations for optimal CAR binder design

**DOI:** 10.1093/abt/tbae003

**Published:** 2024-02-14

This is a correction to: Richard Smith, Bringing cell therapy to tumors: considerations for optimal CAR binder design, *Antibody Therapeutics*, Volume 6, Issue 4, October 2023, Pages 225–239, https://doi.org/10.1093/abt/tbad019

In the originally published version of this manuscript, sections with statements needful for compliance were erroneously omitted.

Sections on **FUNDING, DATA AVAILABILITY, ETHICS AND CONSENT STATEMENT**, and **ANIMAL RESEARCH STATEMENT** were removed from online html/PDF versions. These have been replaced to read:

“**FUNDING** 

Richard Smith is an employee and shareholder of Kite, a Gilead Company.”

“**DATA AVAILABILITY** 

This is not applicable

**ETHICS AND CONSENT STATEMENT** 

This is not applicable

**ANIMAL RESEARCH STATEMENT** 

This is not applicable”

These replacements have been emended in the article.

